# Efficacy of Prompt Initiation of Antiretroviral Therapy in the Treatment of Hemophagocytic Lymphohistiocytosis Triggered by Uncontrolled Human Immunodeficiency Virus

**DOI:** 10.1155/2017/8630609

**Published:** 2017-08-10

**Authors:** Bryan P. Fitzgerald, Amy L. Wojciechowski, Rajinder P. S. Bajwa

**Affiliations:** ^1^Niagara Falls Memorial Medical Center, 621 10th Street, Niagara Falls, NY 14302, USA; ^2^D'Youville College School of Pharmacy, Buffalo, NY 14201, USA

## Abstract

Hemophagocytic lymphohistiocytosis (HLH) is a life-threatening, rapidly progressive hematologic disorder involving uncontrolled immune system activation. HLH has been associated with viral infections, including human immunodeficiency virus (HIV) infections. We report a case of a critically ill 30-year-old female who was hospitalized with HIV-associated HLH, with a CD4 count of 4 cells/mL and HIV viral load of 1,842,730 copies/mL. After ruling out other potential infectious causes of HLH, antiretroviral therapy (ART) was initiated with darunavir, ritonavir, tenofovir, and emtricitabine. Within one week of initiation of ART, the patient began to improve clinically and hematologically and was stable enough for discharge from the hospital three weeks after starting therapy. This case suggests that treatment with ART in patients with HIV-associated HLH should be considered even in critically ill patients with low CD4 counts.

## 1. Introduction

Hemophagocytic lymphohistiocytosis (HLH) is a rare, life-threatening, and rapidly progressive condition caused by excess activation of the immune system. HLH can be precipitated by a variety of triggers, including infection, rheumatologic diseases, or malignancy. Treatment often requires chemotherapy and corticosteroid therapy, along with treating any underlying causes of HLH. Even with standard treatments, prognosis of patients with HLH is poor with approximately 50–60% survival [1, 2]. Herein, we report a case of HLH associated with human immunodeficiency virus (HIV) successfully treated with antiretroviral therapy (ART).

## 2. Case Presentation

A 30-year-old Caucasian female presented to the emergency department with complaints of increasing lethargy and fatigue over the course of several weeks, along with a one-day history of shortness of breath, hematemesis, and palpitations. Past medical history was significant for HIV, anemia, and septoplasty. She was intubated on admission for airway protection due to concern for impending respiratory failure. On exam she was found to be febrile with *T*max of 39.9C, heart rate of 122 beats per minute, respiratory rate of 30 breaths per minute, and blood pressure of 91/57 mmHg, saturating at 97% on 35% FiO_2_. Complete blood count revealed white blood cells of 3 × 10^3^ cells/mL with 73% segs and 15% bands, hemoglobin of 4.5 g/dL, hematocrit of 17.7%, and platelets of 4,000 cells/mL. Ferritin was significantly elevated at 19,406 ng/mL and triglycerides were 412 mg/dL. HIV viral load was 1,842,730 copies/mL and CD4 count was 4 cells/mL. Physical exam revealed hepatosplenomegaly which was confirmed by abdominal ultrasound. The patient was diagnosed with HLH based on meeting the required five of eight criteria set forth in HLH-2004 guidelines (fever > 38.5C, splenomegaly, bicytopenia with hemoglobin < 9 g/dL and platelets < 100,000 cells/mL, triglycerides > 265 mg/dL, and ferritin > 500 ng/mL) [3]. The other three criteria (NK cell activity, CD25 level, and pathological evidence of hemophagocytosis) were not evaluated due to unavailability of labs and thrombocytopenia precluding bone marrow biopsy.

The patient was placed on empirical antibiotics (levofloxacin and vancomycin) for sepsis while investigation for infectious source was conducted. She was also started on methylprednisolone for respiratory failure at admission, which was continued for three days. Blood and urine cultures were negative. Sputum culture grew normal flora. Tests were negative for* Pneumocystis jirovecii*, acid fast bacilli, cytomegalovirus, parvovirus, Epstein-Barr virus, cryptococcus, histoplasmosis, syphilis, and influenza A. The only positive finding was antibody for influenza B, which was attributed to a mild upper respiratory infection that had resolved on its own with supportive measures two weeks prior to admission.

**Figure 1:**
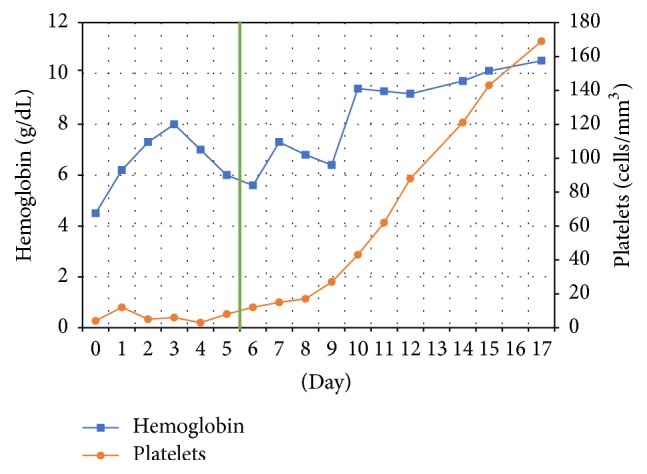
Timeline of hematological response. Vertical line represents initiation of ART.

Despite aggressive transfusions, significant anemia and thrombocytopenia persisted. At this point, the most likely trigger of the HLH was determined to be an uncontrolled HIV infection. Given the patient's clinical instability and unresponsiveness to supportive treatment, ART was empirically initiated with tenofovir/emtricitabine, darunavir, and ritonavir. Within one week of initiation of ART, the patient improved clinically and was able to be extubated, and her laboratory parameters began to normalize (Figure 1). By the time the patient was discharged three weeks after ART initiation, hemoglobin had risen to 10.5 g/dL and platelets to 169,000 cells/mL.

## 3. Discussion

HLH has been associated with viral infections, including Epstein-Barr virus, cytomegalovirus, herpes simplex virus, influenza, and human immunodeficiency virus (HIV) [4–6]. HLH has been reported in patients infected with HIV, with the onset of HLH seen oftentimes during the acute phase of HIV infections [7–9].

Several reports have been published describing patients with HIV-associated HLH who have been successfully treated with ART, with reports of clinical response occurring within five to seven days of initiation of ART. These patients, however, all had CD4 counts that were much higher than our case patient. One case of HIV-associated HLH in a 27-year-old male with a CD4 count of 138 cells/mL was treated with zidovudine, lamivudine, lopinavir, and ritonavir with clinical improvement seen within five days [7]. Another case of HIV-associated HLH was reported in a 35-year-old male with a CD4 count of 100 cells/mL who was treated with zidovudine, lamivudine, and nelfinavir with clinical improvement seen within six days [10]. A third case of HIV-associated HLH in a 48-year-old male with a CD4 count of 98 cells/mL was treated with raltegravir, tenofovir, and emtricitabine with clinical improvement seen within five days [11].

Despite the efficacy of ART treatment in HIV-associated HLH, some case reports have shown that initiation of ART may be ineffective in some patients. A 32-year-old male with HIV-associated HLH with a CD4 count of 100 cells/mL was treated with efavirenz, lamivudine, and tenofovir without clinical improvement after three weeks of ART treatment; clinical improvement was seen within 24 hours of splenectomy [12]. Additionally, the development of reactive HLH has been reported upon initiation of ART in a patient with HIV, possibly as a result of immune reconstitution inflammatory syndrome (IRIS). This patient, however, had a CD4 count of 26 cells/mL, putting him at a greater risk of IRIS than the reported cases successfully treated with ART [13].

In our patient with HIV-associated HLH, despite a very low CD4 count of 4 cells/mL, initiation of ART was not associated with IRIS or other adverse outcomes. Clinical improvement was seen within one week of treatment initiation with ART consisting of darunavir, ritonavir, tenofovir, and emtricitabine. The individual antiretroviral drugs composing the ART regimen are not suspected to be responsible for the clinical improvement of patients with HIV-associated HLH; rather, the initiation of ART to treat the underlying HIV infection is likely responsible. There is currently not enough information to determine if one ART regimen is better than another regimen in the treatment of HIV-associated HLH.

The time to clinical improvement in this patient is consistent with previously reported times to clinical improvement in patients with HIV-associated HLH treated with ART. Prompt treatment with ART has been previously suggested as an effective treatment strategy in patients with HIV-associated HLH [7, 10]. The results of this case report help to reinforce that prompt initiation of ART in HIV-associated HLH as soon as possible once other infectious causes have been ruled out leads to clinical improvement within days of initiation and should be considered as an effective treatment option in these patients. Even in critically ill patients with a very low CD4 count, cautious initiation of ART should be considered if other infectious causes of HLH have been ruled out.
